# Heparin-binding growth factor (HDGF) drives radioresistance in breast cancer by activating the STAT3 signaling pathway

**DOI:** 10.1186/s12967-021-03021-y

**Published:** 2021-08-10

**Authors:** Lingyun Qiu, Yan Ma, Xiaohua Chen, Liheng Zhou, Haibo Zhang, Guansheng Zhong, Lei Zhang, Jianming Tang

**Affiliations:** 1 Oncology Center, Department of Radiation Oncology, Zhejiang Provincial People’s Hospital, Affiliated People’s Hospital, Hangzhou Medical College, Hangzhou, 310014 Zhejiang People’s Republic of China; 2 The First School of Clinical Medicine, Lanzhou University, Lanzhou, 730000 People’s Republic of China; 3 Department of Radiation Oncology, The First Hospital of Lanzhou University, Lanzhou University, Lanzhou, 730000 Gansu People’s Republic of China; 4 Department of Breast Surgery, Renji Hospital, School of Medicine, Shanghai Jiao Tong University, Shanghai, 200127 People’s Republic of China; 5 Department of Breast Surgery, The First Affiliated Hospital, College of Medicine, Zhejiang University, Hangzhou, 310003 People’s Republic of China; 6 Department of Radiation Oncology, Renji Hospital, School of Medicine, Shanghai Jiao Tong University, Shanghai, 200127 People’s Republic of China; 7 Key Laboratory of Biotherapy and Regenerative Medicine of Gansu Province, The First Hospital of Lanzhou University, Lanzhou University, Lanzhou, 730000 Gansu People’s Republic of China

**Keywords:** HDGF, STAT3 signaling pathway, Radioresistance, Breast cancer

## Abstract

Although reports implicate radioresistance as an important obstacle for the management of breast cancer, its molecular mechanism is elusive. Herein, we found that high HDGF levels are expressed significantly in breast cancer and exhibit a positive association with poor survival prognosis. Heparin-binding growth factor (HDGF) was upregulated in radioresistant breast cancer cells, however, its knockdown could reduce breast cancer radioresistant both in vitro and in vivo. Additionally, the binding of RXRα to HDGF promoter blocked HDGF transcriptional activity, consequently inhibiting breast cancer radioresistance. The enhanced radioresistant activity of HDGF is induced by TKT and STAT3, impacting the STAT3-Tyr705 and STAT3-Ser727 phosphorylation and STAT3 transcriptional activity. Notably, HDGF depletion renders radioresistant hypersensitive to the drug that targets STAT3 phosphorylation. This article demonstrates the novel function of HDGF as a promising molecular target for predicting radioresistance in breast cancer.

**Supplementary Information:**

The online version contains supplementary material available at 10.1186/s12967-021-03021-y.

## Introduction

Breast cancer has become one of the cancers with high survival rate. As long as most patients can be detected early and receive standard treatment, the 5-year survival rate is as high as 80%. Nevertheless, breast cancer is still one of the most deadly cancers for women [1, 2]. Diagnosis of breast cancer is more prevalent among women [3, 4]. At present, surgical resection remains the most effective approach for breast cancer management [5]. Notably, conventional radiotherapy is widely administered as an adjuvant therapy post surgery [6]. Recent findings indicate that ionizing radiation (I.R.) influences several gene characteristics, including expression levels, epigenetics, etc., which potentially cause radioresistance [7]. Unsuccessful therapy of breast cancer patients is, in most cases, associated with radioresistance. The therapeutic efficacy is often hampered by the development of radioresistance in breast cancer cells [8]. Hence, uncovering the precise molecular mechanisms that modulate radioresistance is crucial for the clinical management of breast cancer.

Heparin-binding growth factor (HDGF) was first purified from the Huh-7 cell medium, a human hepatoma-derived cell line [9]. Recently, it was found to exert critical functions in vascular development and mitosis [10], and promote malignant processes including cell proliferation, invasion, and metastasis [11–15]. Studies have also revealed the association of HDGF expression with clinical outcomes of patients with pancreatic cancer [16], gastric cancer [17], hepatocellular carcinoma [18]. Nevertheless, the precise role of HDGF in the radioresistance of breast cancer remains largely unknown.

In the present study, we found dramatically upregulated HDGF levels in radioresistant breast cancer cells. Also, we uncovered the role of HDGF in radioresistant breast cancer both in vitro and in vivo *and* explored its underlying molecular mechanism.

## Results

### HDGF is overexpressed in breast cancer and negatively associated with the clinical outcome of patients

**Fig. 1:**
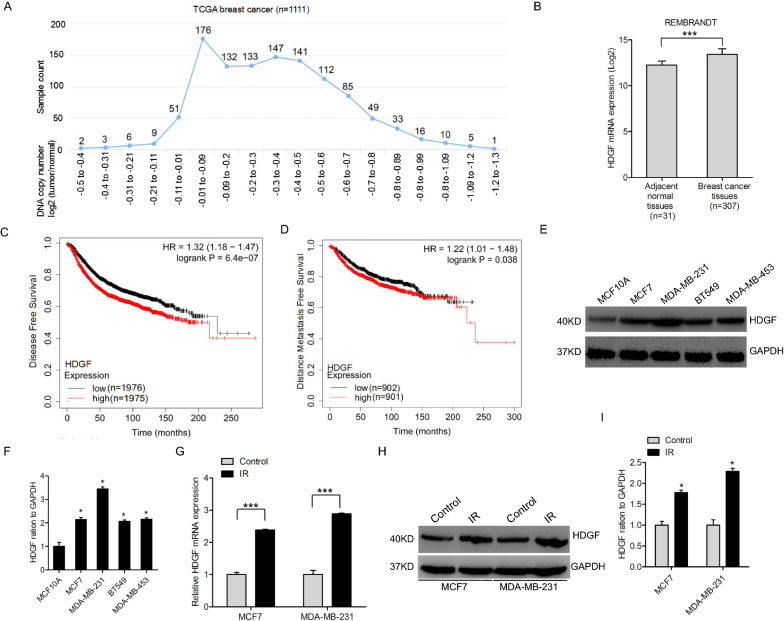
HDGF overexpression in breast cancer and its negative correlation with the survival prognosis of patients. **A** HDGF copy number analysis in TCGA breast cancer dataset. Data were downloaded from http://xena.ucsc.edu/public-hubs. **B** HDGF expression is higher in breast cancer tissues than that in adjacent normal tissues. Expression data of HDGF were downloaded from the REMBRANDT dataset. Data are presented as mean ± SEM.***P < 0.001, by two-tailed t-test. **C**, **D** HDGF expression is negatively correlated with patients' disease-free survival (**C**) and distance metastasis-free survival (**D**). Data were retrieved from Kaplan–Meier Plotter (http:// kmplot.com/ analysis/index). **E** HDGF protein is upregulated in breast cancer (MCF7, MDA-MB-231, BT549, and MDA-MB-453) compared with that in the normal MCF10A cells. **F** Quantification of HDGF protein in **E**. **G**, **H** HDGF is upregulated in breast cancer I.R. cell lines at the mRNA (**G**) and protein level (**H**). **I** Quantification of HDGF protein in (**H**). As shown in **B**, **F**, **G**, and **I**, Error bars ± S.D. *P < 0.05. ***P < 0.001. Data are representative of three independent experiments

To uncover the precise role of HDGF in breast cancer, we first examined the DNA copy number of HDGF from the TCGA database (http://xena.uscs.edu/public-hubs). We found positive HDGF amplification in breast cancer tumors (Fig. 1A). The mRNA expression of HDGF was higher in breast tumor tissues compared to the adjacent normal tissues in the REMBRANDT dataset (http://www.betastasis.com) (Fig. 1B). Moreover, Kaplan–Meier plotter database showed that HDGF expression is negatively correlated with patients' disease-free survival (Fig. 1C) and distance metastasis-free survival (Fig. 1D). Meanwhile, HDGF protein expression in breast cancer (MCF7, BT549, MDA-MB-231, and MDA-MB-453) was higher compared to that in the normal MCF10A cells (Fig. 1E, F). Eventually, we found HDGF expression in radioresistant and control breast cancer cells. Compared to the control cells, the mRNA and protein levels of HDGF were markedly higher in radioresistant breast cancer cells (Fig. 1G–I).

### HDGF enhances radioresistance in breast cancer cells

**Fig. 2:**
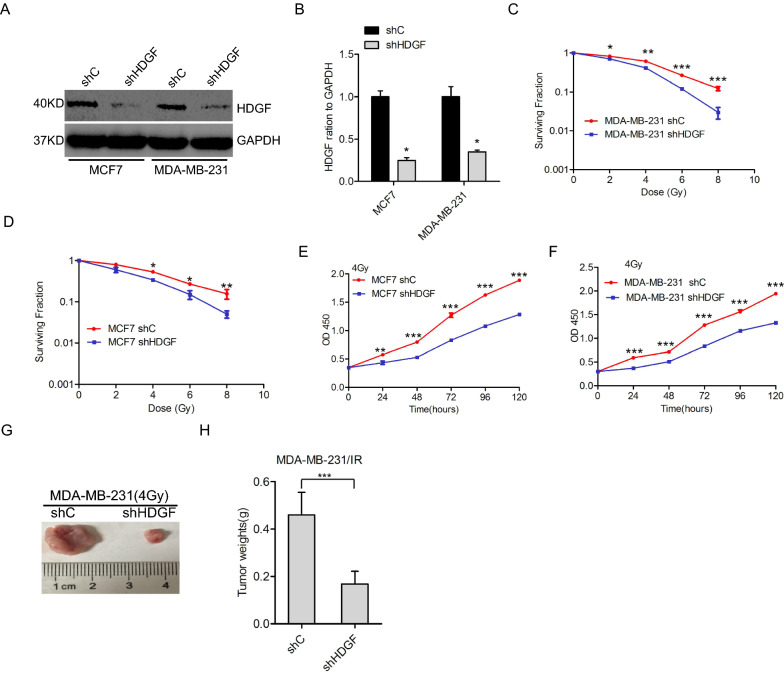
HDGF-enhanced radioresistance of breast cancer cells. **A** Western blot analysis of HDGF protein levels in HDGF knockdown breast cancer cells. **B** Quantification of HDGF protein in (**A**). **C**, **D** NPC cells downregulating HDGF show significantly lower surviving fractions compared to breast cancer cells post-irradiation at 2, 4, 6, 8, and 10 Gy after 2 weeks. **E**, **F**, Breast cancer cells downregulating HDGF show slower proliferation after 4 Gy irradiation at 24, 48, 72, 96, and 120 h. **G**, Representative xenograft tumors of HDGF knockdown-inhibited tumor size in MDA-MB-231/IR cells. **H**, Quantification of tumor size in (**F**). As shown in **B**, **C**, **D**, **E**, **F**, and **H**, Error bars ± S.D. *P < 0.05. ***P < 0.001. Data are representative of three independent experiments

We validated the actual role of HDGF in the radioresistance of breast cancer, shRNA-mediated HDGF knockdown (Fig. 2A, B). HDGF knockdown markedly decreased the cell survival fraction in a dose-dependent manner at 2, 4, 6, 8, and 10 Gy after 2 weeks (Fig. 2C, D). Importantly, our data revealed that HDGF knockdown inhibited cell proliferation after 4 Gy irradiation treatment at 24, 48, 72, 96, and 120 h (Fig. 2E, F). Notably, in vivo tumor growth also demonstrated a similar effect (Fig. 2G, H). Furthermore, we tested that HDGF knockdown increases ROS formation (Additional file 1: Figure S1). These findings indicate the promotor effects of HDGF on the radioresistance of breast cancer cells.

### RXRα negatively modulates HDGF

**Fig. 3:**
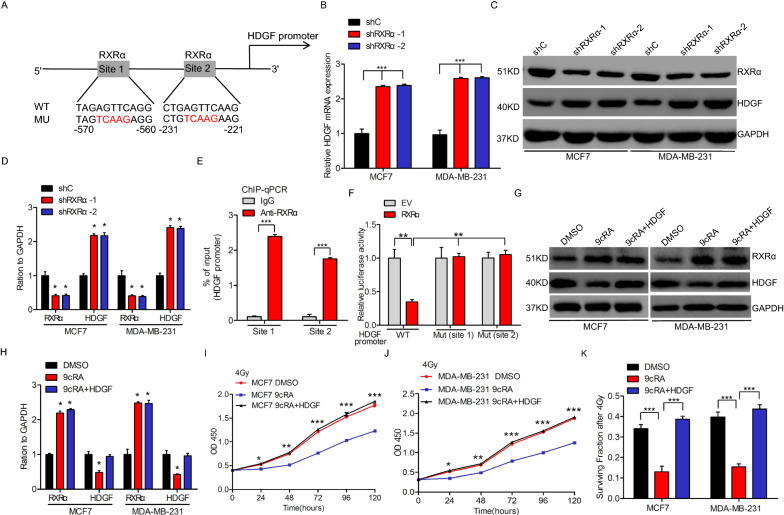
Negative modulation of HDGF by RXRα**. A** Schematic diagram of the putative RXRα binding site in HDGF promoter. **B**, **C** RXRα knockdown inhibited HDGF mRNA (**B**) and protein (**C**) in MCF7 and MDA-MB-231 cells. **D** Quantification of HDGF protein in (**C**). **E** ChIP-qPCR assay of RXRα binding with the HDGF promoter in MDA-MB-231 cells. **F** Luciferase assay of RXRα inhibiting HDGF promoter activity in MDA-MB-231 cells. **G** Effects of 9-cis-retinoic acid (20umol/L) with or without HDGF overexpression co-transfection into MDA-MB-231 cells. **H** Quantification of HDGF protein in (**G**). **I**–**K** HDGF rescued 9-cis-retinoic acid (20umol/L)-inhibited cell proliferation (**I**, **J**) and surviving fractions (**K**) after irradiation. As shown in **B**, **D**, **E**, **F, G**, **H**, **I**, **J**, and **K**, Error bars ± S.D. *P < 0.05. **P < 0.01. ***P < 0.001. Data are representative of three independent experiments

To explore the upstream gene of HDGF, we assessed the transcriptional factors in the JASPAR database. Then, RXRα was selected as one of the most potential candidates that bind to HDGF promoter (Fig. 3A). It was revealed that RXRα knockdown upregulated HDGF mRNA (Fig. 3B) and protein (Fig. 3C, D) expression levels. Further analysis demonstrated that RXRα binds to HDGF promoter at both − 570 to − 560 bp (site 1) and − 231 to − 221 bp (site 2) sites via chromatin immunoprecipitation (ChIP)-qPCR assays (Fig. 3E). RXRα overexpression markedly blocked HDGF promoter transcriptional activity, whereas mutation of site 1 and site 2 induced HDGF promoter transcriptional activity, suppressed by RXRα luciferase assays (Fig. 3F). 9-cis-retinoic acid (9cRA) is an endogenous ligand for RXRα, previously found to potentially enhance cell radioresistance [19, 20]. Similarly, we treated cells with 9cRA or 9cRA, following HDGF overexpression (Fig. 3G, H), and then evaluated the radiation cell proliferation and cell survival fraction. 9cRA markedly blocked the radiation cell proliferation (Fig. 3I, J) and cell survival fraction (Fig. 3K), whereas HDGF overexpression rescued this effect. Therefore, we deduced that HDGF is critical for RXRα-regulated radioresistance.

### HDGF interacts with STAT3 and promotes its transcription activity

**Fig. 4:**
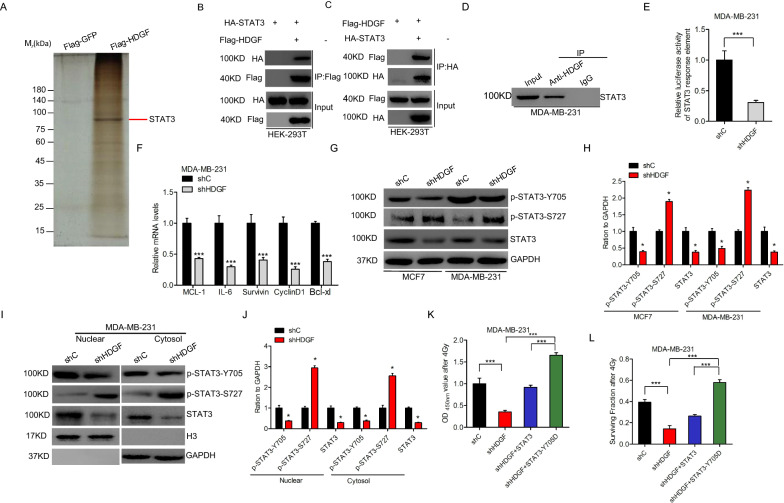
The interaction of HDGF with STAT3 promotes its transcription activity. **A** Immunoprecipitation followed by silver staining of the HDGF binding proteins. **B**, **C** Immunoprecipitation and western blotting for HDGF association with STAT3 protein in HEK-293 T cells. **D** HDGF interacts with STAT3 in MDA-MB-231 cells. **E** Luciferase assay detected STAT3 downstream genes. **F** HDGF knockdown lowers the mRNA levels of STAT3 downstream genes in MDA-MB-231 cells. **G** Effects of STAT3–Tyr705 and STAT3–Ser727 phosphorylation levels in HDGF knockdown breast cancer cells. **H**, Quantification of protein in (**G**). **I** Effects of the cellular distribution of the phosphorylated STAT3–Y705 and STAT3–S727 in HDGF knockdown breast cancer cells. **J** Quantification of protein in (**I**). **K**, **L** Cell proliferation (**K**) and surviving fractions (**L**) for MDA-MB-231 cells transfected with wild–type STAT3, the STAT3–S727D mutant or control after irradiation. As shown in **E**, **F**, **H**, **J**, **K**, and **L**, Error bars ± S.D. *P < 0.05. ***P < 0.001. Data are representative of three independent experiments

For this experiment, we purified the HDGF complex using Flag pull-down from MDA-MB-231 cells overexpressing Flag-tagged HDGF or a Flag-GFP control to reveal the HDGF regulatory mechanism of radioresistance in breast cancer. This was followed by mass spectrometric analysis. The results implicated STAT3 as one of the most potential candidates that bind to HDGF (Fig. 4A). The interaction was validated via immunoprecipitation assays (Fig. 4B–D). HDGF knockdown suppressed the activity of luciferase reporter with the STAT3 response element (Fig. 4E). Furthermore, the mRNA expression of the downstream genes of STAT3 was impeded through HDGF suppression (Fig. 4F). STAT3 phosphorylated modification is critical for nuclear translocation its transcriptional activity [21]. Herein, we detected the effect of HDGF knockdown on STAT3 phosphorylation. Of note, HDGF knockdown suppressed nuclear STAT3 and STAT3-Y705 phosphorylation but promoted nuclear STAT3-Y705 phosphorylation (Fig. 4G–J). The Y705D mutant or STAT3 overexpression rescued HDGF from the inhibition of radiation cell proliferation (Fig. 4K) and cell survival fraction (Fig. 4L). These observations indicate that the transcription activity of HDGF is achieved followed its interaction with STAT3.

### HDGF mediates STAT3 phosphorylation under transketolase

**Fig. 5:**
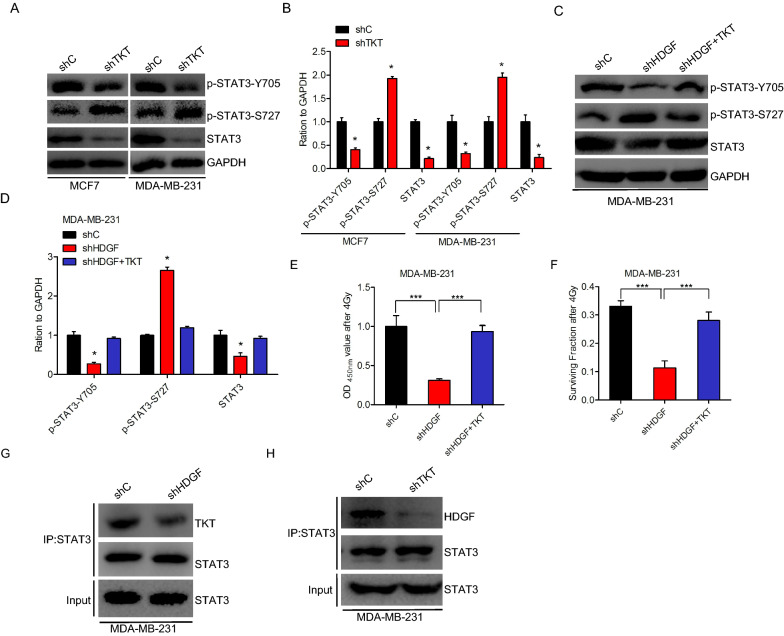
HDGF-mediated STAT3 phosphorylation in combination with transketolase. **A** Effects of STAT3–Tyr705 and STAT3–Ser727 phosphorylation levels in TKT knockdown breast cancer cells. **B** Quantification of protein in (**A**). **C** Effects of TKT rescue HDGF knockdown on STAT3–Tyr705 and STAT3–Ser727 phosphorylation levels. **D** Quantification of protein in (**C**). **E**, **F** TKT overexpression rescue HDGF knockdown-inhibited cell proliferation (**E**) and surviving fractions (**F**) after irradiation. **G** HDGF knockdown inhibits TKT association with STAT3. **H** TKT knockdown inhibits HDGF association with STAT3. As shown in **B**, **D**, **E**, and **F** Error bars ± S.D. *P < 0.05. ***P < 0.001. Data are representative of three independent experiments

Here, we examined the potential mechanism by which HDGF promotes STAT3-Y705 phosphorylation and suppresses nuclear STAT3-Y705 phosphorylation. Transketolase (TKT) was found to regulate STAT3-Y705 and STAT3-S727 phosphorylation in cancers [22, 23]. Our mass spectrometric method identified TKT interaction with HDGF in MDA-MB-231 cells, demonstrating that HDGF potentially mediates STAT3 phosphorylation when combined with TKT. TKT knockdown suppressed STAT3-Y705 phosphorylation and enhanced STAT3-S727 phosphorylation (Fig. 5A–D). These effects were rescued by HDGF overexpression. Furthermore, TKT restored HDGF inhibition-suppressed the radiation cell proliferation (Fig. 5E) and cell survival fraction (Fig. 5F). Interestingly, HDGF knockdown blocked the binding between TKT and STAT3 (Fig. 5G), and less HDGF bound to STAT3 at downregulated TKT levels (Fig. 5H). These results imply that HDGF mediates STAT3 phosphorylation triggered by transketolase.

### HDGF depletion combined with STAT3 activity inhibition impedes breast cancer radioresistance

**Fig. 6:**
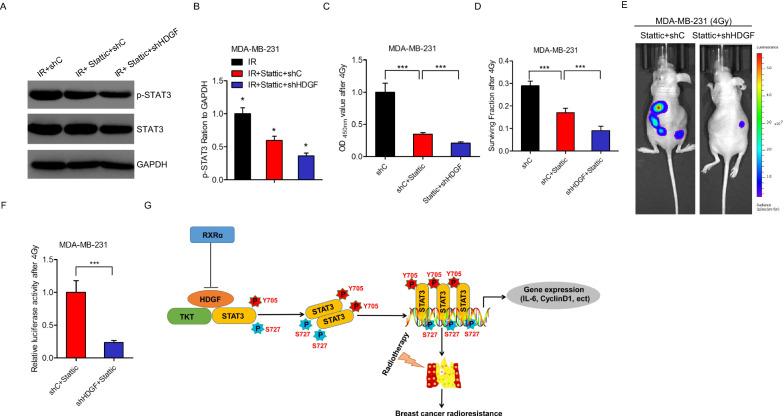
Inhibition of breast cancer radioresistance by HDGF depletion combined with STAT3 activity inhibition. **A**, **C**, **D** Effects of Stattic (20 μmol/L) with or without HDGF knockdown on STAT3 phosphorylation level (**A**), cell proliferation (**C**), and surviving fractions (**D**) after irradiation. **B** Quantification of protein in (**A**). **E** Representative bioluminescence images of Stattic (20 μmol/L) or Stattic (20 μmol/L) combined with HDGF knockdown -transfected MDA-MB-231 cells injected into the mice mammary gland fat pads. Mice were imaged 4 weeks after transplantation. Data were from two independent experiments with 5 mice per group with similar results. **F** Quantification of bioluminescence activity in (**E**). **G** the working model showing that RXRα binds with HDGF promoter and suppresses HDGF transcriptional activity. HDGF could associate with TKT and STAT3, which promotes STAT3 phosphorylation and transcriptional activity, thereby exerts tumor radioresistance in breast cancer. As shown in **B**, **C**, **D**, and **F** Error bars ± S.D. *P < 0.05. ***P < 0.001. Data are representative of three independent experiments

As previously reported, STAT3 activation and dimerization could be inhibited by a small molecular inhibitor (Stattic) [24]. Considering the critical role of the STAT3 signaling pathway in HDGF/TKT-driven breast cancer radioresistance, we assessed the effects of Stattic treatment combined with HDGF-depletion on breast cancer radioresistance using the MDA-MB-231 cells xenograft models. The combined Stattic treatment with HDGF depletion was more effective on the radiation cell proliferation and cell survival fraction compared to single-agent treatment in both breast cancer cell lines (Fig. 6A–D). In vivo assay also found a similar result (Fig. 6E, F). Thus, HDGF depletion combined with STAT3 activity inhibition impedes breast cancer radioresistance.

## Discussion

Studies on the critical roles of HDGF in various cancers, including pancreatic cancer [16], gastric cancer [17], hepatocellular carcinoma [18], have matured. However, there are no reports on the explicit role of HDGF in the radioresistance of breast carcinoma. Herein, we revealed that RXRα binding to the HDGF promoter suppresses HDGF transcriptional activity. The potential association of HDGF with TKT and STAT3 promotes STAT3 phosphorylation and transcriptional activity. In consequence, tumor radioresistance occurs in breast cancer (Fig. 6G).

The extraordinary research team genotyped seven polymorphisms in six genes reported by others as modifiers of oxidative stress (NQO1, mEPXH1, GSTT1 and GSTM1) and inflammation (TNF-α and TGF-β1) for an association in effect of decreasing in liver function tests (LFTs) [25]. In this excellent study, the authors described NQO1, mEPXH1, GSTT1 and GSTM1 play an important role in general oxidative stress defense. Antioxidant therapy play an important role in human health. Free radical participates in DNA damage, induction of apoptosis, and inhibition of growth and proliferation of cancer cells [26]. Antioxidant therapy help to scavenge free radical and might help anticancer therapy such as radiotherapy. High doses of dietary antioxidants (vitamin C, vitamin E succinate and natural beta-carotene) which can be used adjunctively with radiation therapy [27]. Antioxidants may alleviate radiation toxicities [28, 29]. Antioxidant therapy might contribute to human health and plays an important role in the prevention and treatment of diseases. As previously reported, radioresistance is a crucial tumor recurrence factor characterized by the survival fraction [30]. Therefore, to establish the association of HDGF with radioresistance of breast cancer, we prepared the clonogenic assay to assess the survival fraction. Notably, the suppression of HDGF induced radioresistance. HDGF is positively associated with radioresistance in esophageal cancer [31]. Our in vivo experiments demonstrated that HDGF downregulation inhibited breast cancer radioresistance, suggesting its potential association with breast cancer radioresistance.

Recent reports show that RXRα is critical for breast cancer progression and has been proven to be a transcriptional factor inducing tumor suppression in breast cancer [32–34]. Interestingly, RXRα was reported to inhibit radioresistance in the Head and Neck Squamous Cell Carcinoma [35]; however, its explicit role in breast cancer remains largely elusive. Our findings demonstrated the direct association of RXRα with HDGF promoter and that it negatively mediates HDGF transcriptional activity. Furthermore, RXRα agonist, 9cRA, rescued HDGF overexpression-increased the survival fraction and cell proliferation after I.R. Taken together, the present findings demonstrate that HDGF is critical in RXRα suppression of breast cancer radioresistance.

Activated STAT3 is a potential molecular target in the management of numerous cancers [36–39]. STAT3 signaling is involved in the regulation of metastasis, the transition of cancer stem cells, and chemoresistance of cancer by epithelial-mesenchymal transition [40]. STAT3 have been reported to localize to mitochondria. The mitochondrial localization of STAT3 is required for its ability to support malignant transformation in breast cancer cells [41]. Some genes contribute to the alteration of STAT3 phosphorylation status, consequently influencing its nuclear import–export dynamics [42, 43]. The association of Lnc-DC with STAT3 promotes the Y705 phosphorylation [42]. Herein, we found the HDGF binding with STAT3 could promote the Y705 phosphorylation, thereby inducing STAT3 transcriptional activity. A recent study revealed that activated STAT3 is related to Y705 and/or S727 phosphorylation [22]. Y705 show oncogenic characteristic in several cancers [44]. S727 phosphorylation was found to potentially induce or suppress Y705-phosphorylated STAT3 [45]. In the present study, we demonstrated that HDGF promoted Y705 phosphorylation and decreased S727 phosphorylation; these effects increased the survival fraction and cell proliferation post I.R. These findings affirm the role of HDGF in breast cancer radioresistance through modulation of STAT3 phosphorylation.

TKT, a ubiquitous enzyme, has potential catalytic effects on the reversible transfer of two-carbon ketolunits between ketose and aldose phosphates, tuning the carbon flow via the non-oxidative branch of the PPP [43]. In hepatocellular carcinoma, TKT exerts an inhibitory effect on STAT3-S727 phosphorylation and activator effect on STAT3-Y705 phosphorylation, respectively [24]. Herein, found that TKT interacted with STAT3 or HDGF. TKT suppression blocked HDGF interaction with STAT3. Additionally, TKT exerted an inhibitory effect on STAT3-S727 phosphorylation and an activator effect on STAT3-Y705 phosphorylation in breast cancer, which rescued HDGF inhibition- suppressed the survival fraction and cell proliferation after I.R. These observations present a novel molecular link between HDGF, STAT3 phosphorylation, and TKT in breast cancer.

In conclusion, the present findings demonstrate that RXRα binds with HDGF promoter and suppresses HDGF transcriptional activity. The potential association of HDGF with TKT and STAT3 promotes STAT3-Y705 phosphorylation and inhibits STAT3-S727 phosphorylation enhancing STAT3 transcriptional activity, thereby increasing tumor radioresistance in breast cancer. Our results reveal the critical roles of the HDGF-TKT-STAT3 signaling pathway in breast cancer radioresistance; thus, it is a promising therapeutic molecular target for breast cancer.

## Materials and methods

### Cell lines

MCF7, BT549, MDA-MB-231, MDA-MB-453, and MCF10A were acquired from the Shanghai Institute for Biological Sciences (Chinese Academy of Sciences, Shanghai, China). All cells were cultured in a humidified incubator at 37 °C and 5% CO_2._ Breast cancer cells were cultured in RPMI-1640 (Invitrogen, Carlsbad, CA) supplemented with 10% fetal bovine serum (FBS) (Invitrogen, Carlsbad, CA). MCF10A cells were cultured in DMEM/F12 (Invitrogen, Carlsbad, CA) supplemented with penicillin- streptomycin (100 μg/ml), cholera toxin (100 ng/ml), insulin (10 μg/ml), hydro-cortisone (0.5 μg/ml), epidermal growth factor (20 ng/ml), and horse serum (5%).

### Plasmids

TKT and HDGF plasmids were purchased from Shanghai Bioegene Co., Ltd. The shRNAs were designed as follows: shRXRα-1 (5’-GGCAAGCACTATGG AGTGTAC-3’); shRXRα-2 (5’-TGCGCTCCATCGGGCTCAAAT-3’); shTKT(5’-GCCAT CATCTATAACAACAAT-3’); shHDGF(5’-CGAGAACAACCCTACTGTCAA-3’).

### RNA extraction and qRT-PCR

Total RNA was extracted using Trizol reagent (Takara, Dalian, China). All cDNAs were synthesized using the Reverse Transcription Kit (Takara, Dalian, China). qPCR reactions using the qPCR Master Mix (SYBR Green) (Clontech, USA), with GAPDH as a control. Specific primers are listed in Additional file 2: Table S1.

### Western blot analysis

WB analyses were undertaken following our previously described protocol [46], using the following antibodies: TKT (ab112997, 1:1000, Abcam), HDGF (ab244485, 1:1000, Abcam), RXRA (21218-1-AP, 1:1000, Proteintech), STAT3 (ab119352, 1:1000, Abcam); STAT3 (phospho Y705) (ab76315, 1:1000, Abcam); STAT3 (phospho S727) (ab86430; 1:1000; Abcam); GAPDH (ab8245; 1:5000; Abcam).

### ChIP-qPCR

ChIP was performed using ChIP Kit (Millipore-17-408) following the manufacturer's protocol. Purified ChIP DNA was subjected to qRT-PCR. All primers are listed in Additional file 2: Table S1.

### Luciferase promoter assay

pGL3-HDGF promoter plasmids were prepared for co-transfection with RXRA or empty vector using the Lipofectamine 3000 transfection reagent (Invitrogen). pRL Renilla luciferase vector (Promega) acted as a control group. A dual-luciferase Reporter kit (Promega) was employed to detect the luciferase signals.

### LC–MS/MS analysis

Tryptic peptides = dissolved in 0.1% formic acid (solvent A) were directly loaded onto a custom-made reversed-phase analytical column (15-cm length, 75 μm i.d.). The gradient depicted an increase from 6 to 23% solvent B (0.1% formic acid in 98% acetonitrile) over 16 min, 23% to 35% in 8 min before rising to 80% in 3 min, and then holding at 80% for the last 3 min. The peptides were subjected to an NSI source. This was followed by tandem mass spectrometry (MS/MS) in Q ExactiveTM Plus (Thermo) coupled online to the UPLC. We applied an electrospray voltage at2.0 kV. The m/z scan ranged between 350 and1800 for a full scan. Intact peptides were detected in the Orbitrap at 70,000 resolution. Then, peptides were selected for MS/MS, with the NCE set at 28. The fragments were detected in the Orbitrap at a resolution of 17,500. We performed a data-dependent procedure that alternated between one M.S. scan, followed by 20 MS/MS scans with 15.0 s dynamic exclusion. The automatic gain control (AGC) was set at 5E4.

### Cell proliferation and colony formation

The proliferation of cells seeded in 96-well plates was detected using a WST-1 Assay Kit (Roche). For colony formation, we seeded cells into the 6-well plates, after which cell colonies were stained with 1% crystal violet solution. We recorded the scores and analyzed colony counts.

### Tumorigenesis studies

Four-weeks old female athymic NCr-nu/nu mice (SLAC, Shanghai, China) were categorized into two groups randomly. Each group comprised five mice. Subsequently, MDA-MB-231 cell suspension (5 × 10^6^) was injected into mammary fat pads of mice. Approval for animal experiments was issued by the Guidance of Institutional Animal Care and Use Committee (IACUC) from Zhejiang Provincial People's Hospital. The IVIS Lumina imaging station (Caliper Life Sciences) was adopted for bioluminescence imaging.

### Statistical analysis

The clonogenic survival assay was subjected to a one-way analysis of variance. A two-tailed paired Student's t-test was applied to evaluate the significance of data for two groups. A P value < 0.05 denoted statistical significance. The SPSS13.0 software was employed to analyze all statistical data.

## Supplementary Information

**Additional file 1: Figure S1.** Intracellular ROS production probe 2 0,7 0dichlorodihydrofluorescein diacetate (DCF-DA). HDGF knockdown significantly increased ROS formation compared to control group. The data were quantitated, and the results are expressed as the means AE SE.

**Additional file 2: Table S1.** Primers for qRT-PCR assays.

## Data Availability

The datasets used during the current study are available from the corresponding author on reasonable request.
