# Supplementary material for: Heparin-binding growth factor (HDGF) drives radioresistance in breast cancer by activating the STAT3 signaling pathway
Source: J Transl Med. 2021 Aug 10;19:344. doi: 10.1186/s12967-021-03021-y (PMC8353798; doi:10.1186/s12967-021-03021-y)
Supplement: Supplementary file 1 — Additional file 1: Figure S1. Intracellular ROS production probe 2 0,7 0dichlorodihydrofluorescein diacetate (DCF-DA). HDGF knockdown significantly increased ROS formation compared to control group. The data were quantitated, and the results are expressed as the means AE SE. [file 12967_2021_3021_MOESM1_ESM.pdf]

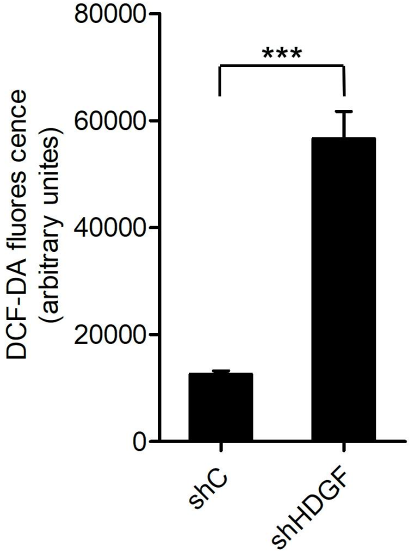

Supplementary Figure 1 Intracellular ROS production was measured using the peroxide-sensitive fluorescent probe 2 0 ,7 0dichlorodihydrofluorescein diacetate (DCF-DA). HDGF knockdown significantly increased ROS formation compared to control group. The data were quantitated, and the results are expressed as the means AE SE.
