# Supplementary material for: Heparin-binding growth factor (HDGF) drives radioresistance in breast cancer by activating the STAT3 signaling pathway
Source: J Transl Med. 2021 Aug 10;19:344. doi: 10.1186/s12967-021-03021-y (PMC8353798; doi:10.1186/s12967-021-03021-y)
Supplement: Supplementary file 2 — Additional file 2: Table S1. Primers for qRT-PCR assays. [file 12967_2021_3021_MOESM2_ESM.docx]

**Table S1** Primers for qRT-PCR assays

| Primer pairs | Sequence |
| --- | --- |
| qRT-PCR,GAPDH | 5’-GGAGCGAGATCCCTCCAAAAT-3’ and  5’-GGCTGTTGTCATACTTCTCATGG-3’ |
| qRT-PCR,HDGF | 5’-CTCTTCCCTTACGAGGAATCCA-3’ and  5’-CCTTGACAGTAGGGTTGTTCTC-3’ |
| qRT-PCR,TKT | 5’-TCCACACCATGCGCTACAAG-3’ and  5’-CAAGTCGGAGCTGATCTTCCT-3’ |
| ChIP-PCR,HDGF Site 1 | 5’-TTCTATGCCTCTGTTGTTAGT-3’ and  5’-ATATGCAAAGTCTCCCTGCAA-3’ |
| ChIP-PCR,HDGF Site 2 | 5’-ATGTGGGCAAATACAGTGACA-3’ and  5’-GGCTTCACAAAGGTGATGACT-3’ |
